# KIFCI, a novel putative prognostic biomarker for ovarian adenocarcinomas: delineating protein interaction networks and signaling circuitries

**DOI:** 10.1186/1757-2215-7-53

**Published:** 2014-05-12

**Authors:** Shrikant Pawar, Shashikiran Donthamsetty, Vaishali Pannu, Padmashree Rida, Angela Ogden, Nathan Bowen, Remus Osan, Guilherme Cantuaria, Ritu Aneja

**Affiliations:** 1 Department of Biology, Georgia State University, Atlanta, GA 30303, USA; 2 Center for Cancer Research and Therapeutic Development (CCRTD), Clark Atlanta University, Atlanta, GA 30314, USA; 3 Department of Mathematics and Statistics, Georgia State University, Atlanta, GA 30303, USA; 4 Northside Hospital Cancer Institute, Atlanta, GA 30342, USA

## Abstract

**Background:**

Amplified centrosomes in cancers are recently garnering a lot of attention as an emerging hub of diagnostic, prognostic and therapeutic targets. Ovarian adenocarcinomas commonly harbor supernumerary centrosomes that drive chromosomal instability. A centrosome clustering molecule, KIFC1, is indispensable for the viability of extra centrosome-bearing cancer cells, and may underlie progression of ovarian cancers.

**Methods:**

Centrosome amplification in low- and high- grade serous ovarian adenocarcinomas was quantitated employing confocal imaging. KIFC1 expression was analyzed in ovarian tumors using publically-available databases. Associated grade, stage and clinical information from these databases were plotted for KIFC1 gene expression values. Furthermore, interactions and functional annotation of KIFC1 and its highly correlated genes were studied using DAVID and STRING 9.1.

**Results:**

Clinical specimens of ovarian cancers display robust centrosome amplification and deploy centrosome clustering to execute an error-prone mitosis to enable karyotypic heterogeneity that fosters tumor progression and aggressiveness. Our in silico analyses showed KIFC1 overexpression in human ovarian tumors (n = 1090) and its upregulation associated with tumor aggressiveness utilizing publically-available gene expression databases. KIFC1 expression correlated with advanced tumor grade and stage. Dichotomization of KIFC1 levels revealed a significantly lower overall survival time for patients in high KIFC1 group. Intriguingly, in a matched-cohort of primary (n = 7) and metastatic (n = 7) ovarian samples, no significant differences in KIFC1 expression were detectable, suggesting that high KIFC1 expression may serve as a marker of metastases onset. Nonetheless, KIFC1 levels in both primary and matched metastatic sites were significantly higher compared to normal tissue . Ingenuity based network prediction algorithms combined with pre-established protein interaction networks uncovered several novel cell-cycle related partner genes on the basis of interconnectivity, illuminating the centrosome clustering independent agenda of KIFC1 in ovarian tumor progression.

**Conclusions:**

Ovarian cancers display amplified centrosomes, a feature of aggressive tumors. To cope up with the abnormal centrosomal load, ovarian cancer cells upregulate genes like KIFC1 that are known to induce centrosome clustering. Our data underscore KIFC1 as a putative biomarker that predicts worse prognosis, poor overall survival and may serve as a potential marker of onset of metastatic dissemination in ovarian cancer patients.

## Introduction

Centrosome amplification (CA) is a hallmark of cancers [1-3]. Recent evidence suggests that amplified centrosomes can drive malignant transformation [4] and perhaps fuel metastatic dissemination [2]. Logically, excess centrosomes would orchestrate a multipolar spindle which might result in inviable progeny and jeopardize the survival of extra centrosome bearing cancer cells. Cancer cells however, overcome this paradoxical situation in their favor through multiple mechanisms including centrosome clustering, a cancer cell-specific trait [5-7]. It is being revealedthat cancer cells have indeed evolved quite a sophisticated and extensive arsenal of ‘clever tactics’ to hijack cellular mechanisms and deploy them to cluster supernumerary centrosomes into two polar groups to allow formation of a pseudo-bipolar mitotic spindle [6-9]. A centrosome clustering molecule, KIFC1 (also known as HSET), a minus end-directed motor protein of the kinesin-14 family, is essential for the viability of extra centrosome-bearing cancer cells [1].

Recently, supernumerary centrosomes and high KIFC1 expression have been associated with chromosome missegregation that results in low-grade aneuploidy, a landmark of cancers [6-9]. Given that normal cells most often have two centrosomes, they do not rely on centrosome clustering mechanisms; as a result, targeting KIFC1 is an attractive chemotherapeutic strategy [10]. Proof-of-concept comes from the recent discovery and preclinical development of two novel KIFC1 small molecule inhibitors AZ82 [10] and CW069 [11], that cause centrosome declustering exclusively in cancer cells with amplified centrosomes [10]. Given the excellent promise of KIFC1 as a therapeutic target, its role as a negative prognosticator merits investigation in cancers with amplified centrosomes. Since KIFC1 has been shown to predict non-small cell lung cancer metastasis to the brain [12], we were inquisitive to examine its usefulness as a risk predictor and/or negative prognosticator in other aggressive cancer types which harbor amplified centrosomes. Although centrosomal aberrations in epithelial ovarian cancers (EOC) have been a relatively understudied area, abnormalities in centrosomes have been reported in ovarian tumors [13]. Centrosomal aberrations may be an early event in ovarian carcinogenesis and implicated in ovarian tumor progression. The features of many epithelial tumors, including EOC, are the presence of aneuploidy, a consequence of chromosomal instability (CIN) that arises due to aberrant CA [14]. In several solid malignancies, amplified centrosomes are a potential indicator of cancer aggressiveness [15].

Herein we examined the severity and extent of centrosome amplification in low- and high- grade serous ovarian adenocarcinomas employing multicolor immunofluorescence confocal imaging. In these clinical specimens, we also visualized various cell cycle stages and spindle architecture of cells in mitosis to gain insights into the propensity of ovarian tumors to undergo aberrant cell divisions that foster CIN, karyotypic heterogeneity and generation of aneuploid clones. Further, we quantitated the extent of spindle polarity, in particular, cells with multipolar spindle configurations as well as cells with pseudo-bipolar spindles with centrosomes corralled at the two poles. Given the link between presence of excess centrosomes and upregulation of KIFC1, an important member of the centrosome clustering arsenal, we evaluated KIFC1’s potential as a negative prognostic indicator in EOC. Using independent gene expression datasets, we identified that KIFC1 gene in ovarian cancer is expressed at least 2-fold (logarithm to base 2 scale) higher than in normal ovaries. Our in silico data also suggest a correlation between KIFC1 and grade, stage, and clinical outcomes in EOC. The gene expression profiling-based identification of KIFC1 as a negative prognosticator in EOC may improve evaluation of disease course. In addition, an in silico-guided mechanistic understanding of KIFC1 gene interactions have delineated pathways and protein interactions which illuminate previously unrecognized partners of this centrosome clustering molecule to unravel the biological behavior of ovarian tumors.

## Results

### Epithelial ovarian cancers harbor amplified centrosomes

**Figure 1:**
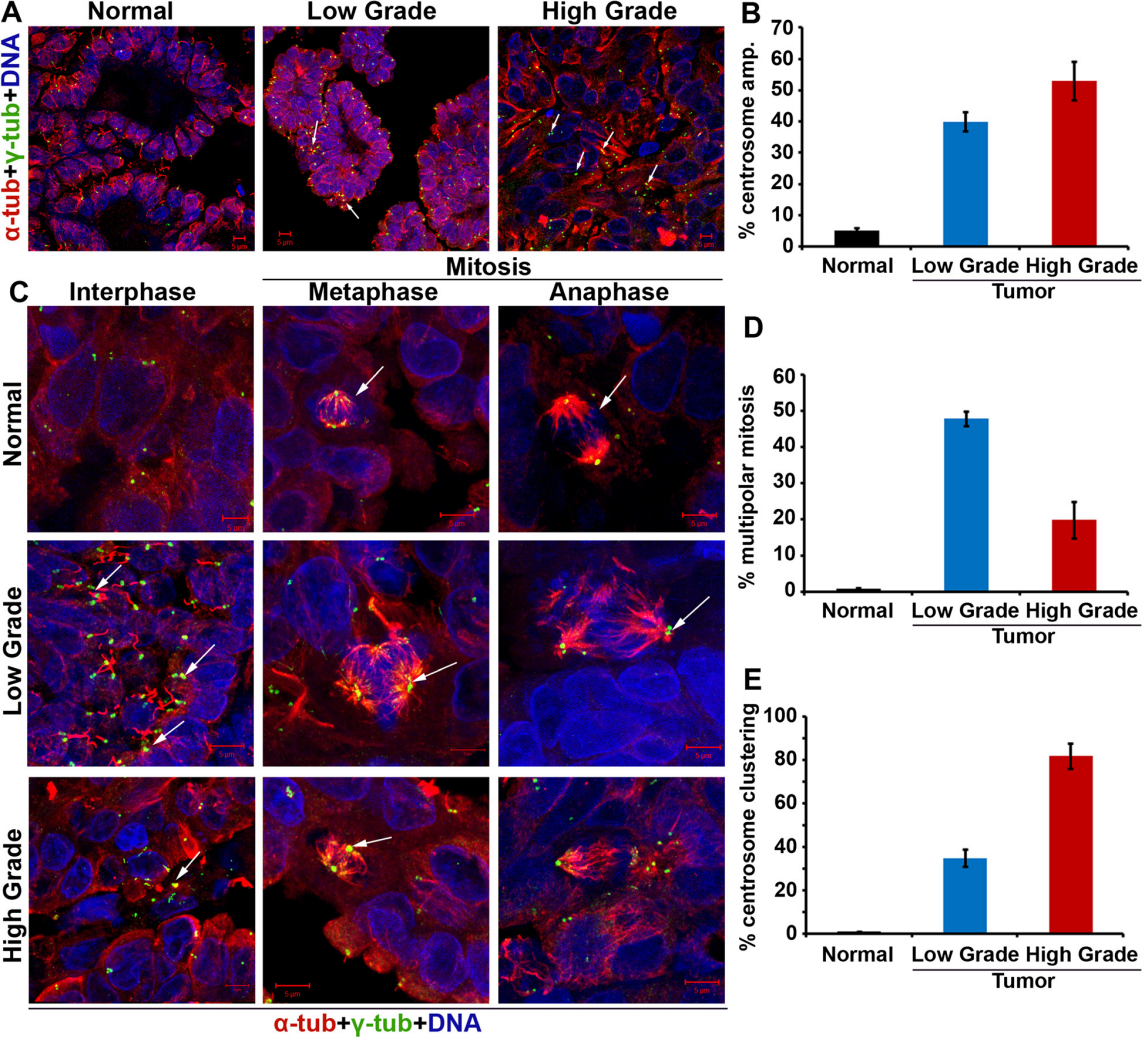
**Centrosome amplification in ovarian cancer.** Ovarian cancer and normal adjacent tissue were stained with α-tubulin (red), γ-tubulin (green), and DAPI (blue) to visualize microtubules, centrosomes, and DNA, respectively. **A**. Confocal micrographs representing centrosome amplification status in ovarian normal, low-grade cancer and high-grade cancer tissue. **B**. Bar graph representation of percent cells showing centrosome amplification in ovarian and normal adjacent tissue. 500 cells were counted in each sample. **C**. Confocal microscopic images represent cells throughout the sequential stages of the cell cycle. Arrows indicate presence of centrosome clustering. Scale bar, 5 μm. **D**, **E**. Bar graph representation of percent cells showing multipolar mitosis and centrosome clustering in ovarian and normal adjacent tissue. 500 cells were counted in each sample. p < 0.05.

Previous studies have reported a strong correlation between aneuploidy and CA in ovarian cancers, with near-tetraploid tumors displaying a higher intratumoral CA, near-diploid tumors showing infrequent centrosomal abnormalities [13,14]. Thus, we first evaluated the extent and severity of CA in a grade-wise manner in ovarian adenocarcinomas. To this end, we examined immunostained centrosomes and mitotic spindles in cells from paraffin-embedded clinical cancer samples derived from low- (n = 7) and high-grade (n = 7) serous ovarian carcinomas. Both low- and high- grade tissues showed CA, with high-grade tumors exhibiting notably higher numerical and structural centrosome aberrations (~50%, indicated with white arrows) as compared to low-grade tumors (~40%, indicated with white arrows). Amplified centrosomes were not observed in any of the normal adjacent tissues, underscoring this anomaly as a tumor-specific one (Figure 1A, B).

### Epithelial ovarian cancers exhibit mitotic aberrations and abnormal spindle architecture

Given the notable differences in centrosomal aberrations between low and high grade ovarian cancers, we next asked if differences in centrosomal aberrations between tumor grades translated into differences in mitotic and spindle aberrations. We found that low- and high- grade tumors significantly differed in the proportion of cells harboring aberrant mitotic spindles. Over 50% mitotic cells in low-grade ovarian tumors exhibited multipolar spindles in stark contrast to a mere 20% multipolar mitotic cells in high-grade tumors (Figure 1C, D). Intriguingly, multipolar spindles observed in both low- and high- grade tumors were predominantly tripolar, suggesting the enabling role of spindle multipolarity in promoting chromosome missegregation that underlies low-grade aneuploidy as opposed to mitotic catastrophe.

### Epithelial ovarian cancers display clustering of amplified centrosomes

The discordance observed in the extent of CA and multipolar mitosis in high-grade tumors naturally led us to hypothesize that high-grade ovarian tumors manage their “excess baggage” of supernumerary centrosomes by clustering them into “pseudobipolar” spindles. Thus we evaluated the extent of centrosome clustering by counting the number of mitotic cells harboring “pseudo-bipolar” spindles. Intriguingly, our observations revealed that 80% of the mitotic cells in high-grade tumors display centrosome clustering compared to only 20% in low-grade tumors (Figure 1C, E). Taken together, our data suggest that ovarian cancers display a high degree of CA which is grade-dependent. Supernumerary centrosomes drive the assembly of multipolar spindle during mitosis to facilitate higher degree of chromosomal heterogeneity, mostly numerical in low-grade tumors. However, high-grade tumors tend to cluster their extra centrosomes and form a “pseudobipolar” spindle to enable low-level of chromosome missegregation as compared to low-grade tumors.

### KIFC1 gene expression level is high in ovarian cancers compared to uninvolved normal ovarian tissue

**Figure 2:**
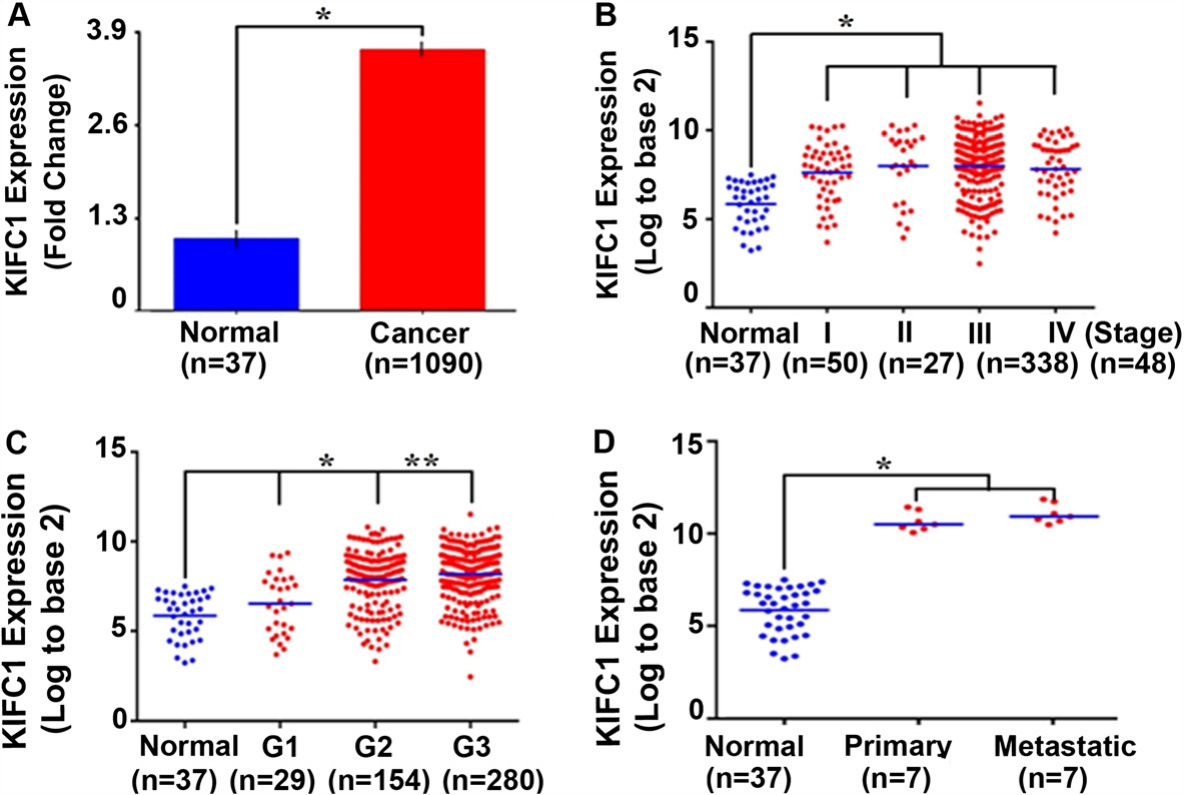
**KIFC1 expression in ovarian cancer and normal tissue. A)** Comparison of KIFC1 expression levels in ovarian cancer and normal ovarian samples. A standard error plot for one channel data comparing fold change for cancer (n = 1090) and normal samples (n = 38) (p value < 0.001). **B)** Box whisker plot for comparing KIFC1 expression in ovarian cancer patients considering stages (n = 468) (p < 0.0001) **C)** Box whisker plot for comparing KIFC1 expression in ovarian cancer patients considering grades (n = 468). **D)** Box whisker plot for comparing KIFC1 expression in primary (n = 7) vs metastatic ovarian cancer (n = 7) (p < 0.05 between Grade 1 and 2 and between Grade 2 and 3).

Having identified a high degree of centrosomal clustering in ovarian cancers, we reasoned that these cells may rely heavily on KIFC1, a centrosome clustering molecule. Thus, we were inquisitive to evaluate the expression of KIFC1, a well known clustering molecule, which is highly expressed in aggressive breast cancers, particularly the triple negatives [10]. To this end, we examined single channel microarray data from GEO and TCGA [16,17] databases to compare KIFC1 gene expression levels in serous ovarian carcinoma to normal ovarian tissue. A fold change for the means of Mas5.0 normalized intensity values in ovarian cancer (n = 1090) over normal ovarian samples (n = 38) revealed an approximately two fold difference, with an average KIFC1 expression value of ~5.86 for normal tissue and ~7.72 for tumors (Figure 2A) (p < 0.001).

### KIFC1 gene expression increases with grade in ovarian cancers

Having found that KIFC1 expression was higher in EOC as compared to normal ovarian tissue, we next assessed if KIFC1 expression changes with grade and stage within EOC. Ovarian carcinoma is categorized by four stages, ranging from stage I in which the cancer is localized to the ovary/ovaries to stage IV in that the cancer has spread outside of the peritoneal cavity [18]. Grades for ovarian carcinoma are classified based on their histological appearance. Grade 1 (well differentiated, grade 2 moderately differentiated, and grade 3 poorly differentiated) [18,19]. Based on 468 EOC samples, average KIFC1 expression levels were ~6.53 for grade 1, ~7.85 for grade 2, and ~8.25 for grade 3, which were significantly different (p < 0.01) amongst these sub-grades (Figure 2C). As a result, higher KIFC1 is associated with increased grade. Average KIFC1 expression levels were approximately 7.62 for stage I, 8.00 for stage II, 8.10 for stage III, and 7.82 for stage IV, and these differences were not statistically significant amongst these sub-stages (Figure 2B). Although we found a significant correlation between KIFC1 expression levels within sub-grades, such differences were not observed within sub-stages. Given our finding that KIFC1 expression increases with grade, we were next interested in determining whether it is expressed at higher levels in metastatic versus primary ovarian carcinomas (Figure 2D). Although both primary and metastatic tumors showed significantly higher expression of KIFC1 as compared to normal tissue, there was no significant difference in the expression value of KIFC1 between matched primary and metastatic ovarian carcinomas (Figure 2D). These fourteen matched sets of primary and metastatic (omental) samples were collected from seven advanced staged (III/IV) ovarian cancer (serous adenocarcinoma) patients and were a part of a recently published paper from John McDonald’s group [20].

### Increased KIFC1 expression is associated with poorer overall survival in age-specific ovarian cancer patients

**Figure 3:**
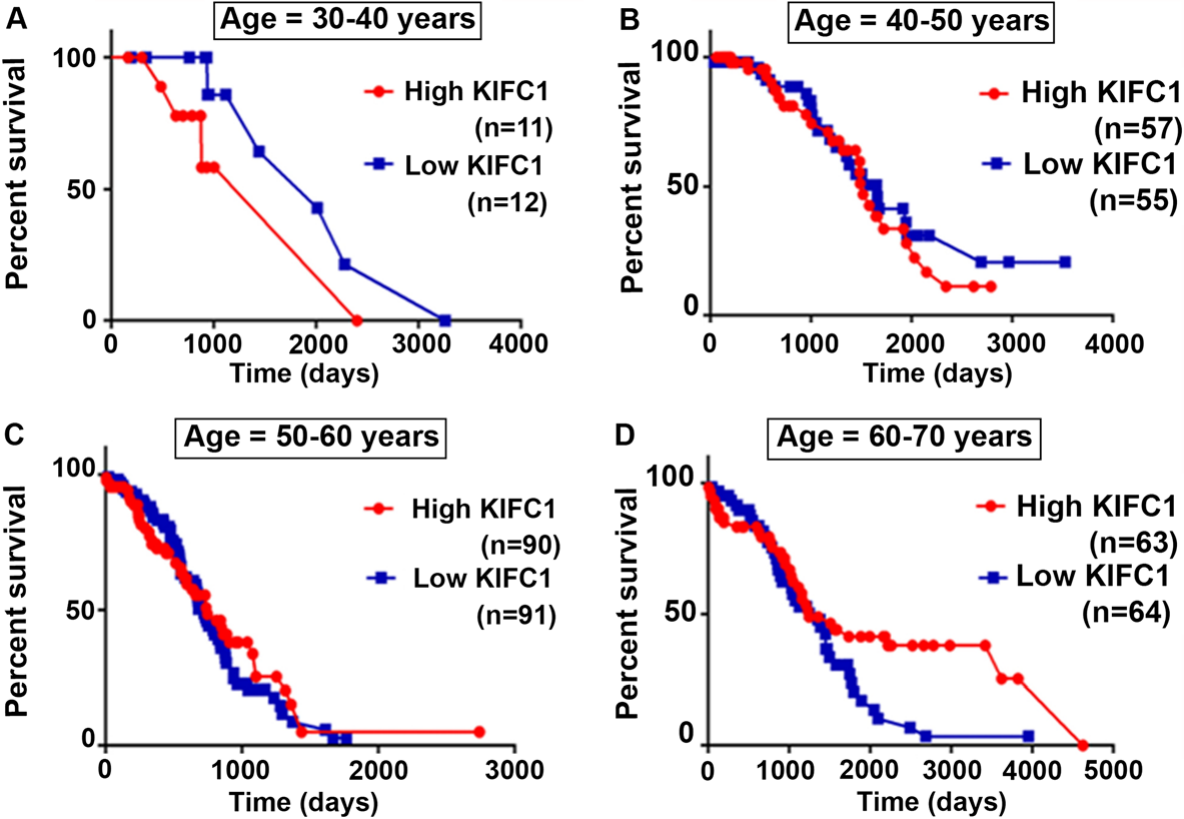
**Overall Survival (OS) plot for high and low KIFC1 groups.** Patient ages range from 30 to 80 years, each group divided with a decade difference. Further subgroups of high and low KIFC1 were made based on averages of KIFC1 expression levels in each group. **A)** OS plot for patients with age group 30 to 40 years. **B)** OS plot for patients with age group 40 to 50 years. **C)** OS plot for patients with age group 50 to 60 years. **D)** OS plot for patients with age group 60 to 70 years. For all the graphs p>0.05.

Studies have shown that five year survival for stage I ovarian cancer is 92%, stage II 55.1%, stage III 21.9%, and 5.6% for stage IV [21] stating a continuous decrease in patient survival rate with increasing cancer stage. Given the association between age, stage, and overall survival, we were keen to investigate the association of KIFC1 expression with these variables. Utilizing datasets from TCGA, we analyzed overall survival in months for patients diagnosed with ovarian cancer. Patients were categorized into six groups by age (30–39, 40–49, 50–59 and 60–69). Furthermore, each age group was divided into two subgroups based on average KIFC1 expression (i.e., low and high). Patients with KIFC1 expression levels more than average KIFC1 expression were categorized as high subgroup and patients with KIFC1 expression levels less than average KIFC1 expression were categorized as low subgroup. Overall survival in days for these patients is plotted in Figure 3, with averages given in Table 1. In patients aged 30–39, the subgroup with low KIFC1 expression had a ~20% increase in survival compared to the high expression subgroup (Figure 3A). In patients aged 40–49 the subgroup with low KIFC1 expression had a ~9% increase in survival compared to the subgroup with high KIFC1 expression (Figure 3B). In patients aged 50–59 years, the subgroup with low KIFC1 expression had a ~5.6% increase in survival compared to the subgroup with high KIFC1 expression (Figure 3C). However, this trend was reversed in patients aged 60 and older possibly due to associated co-morbidities. In these patients, the subgroup with low KIFC1 expression had a ~11% decrease in survival compared to the subgroup with high KIFC1 expression (Figure 3D). Thus, increased KIFC1 expression correlated with poor overall survival in ovarian cancer patients.

**Table 1:** Comparing survival in days for patients with ovarian cancer categorized in different age groups and with high and low KIFC1 expression levels

**Age groups (Years)**	**Survival (days) for high KIFC1 expression**	**Survival (days) for low KIFC1 expression**
30-40	831.7 (N = 11)	1262.2 (N = 12)
40-50	916.2 (N = 57)	1100.2 (N = 54)
50-60	1025.1 (N = 89)	1147.3 (N = 103)
60-70	1239.4 (N = 61)	975.9 (N = 64)

### Interactions and functional annotation of KIFC1 and its highly correlated genes using DAVID and STRING 9.1

**Figure 4:**
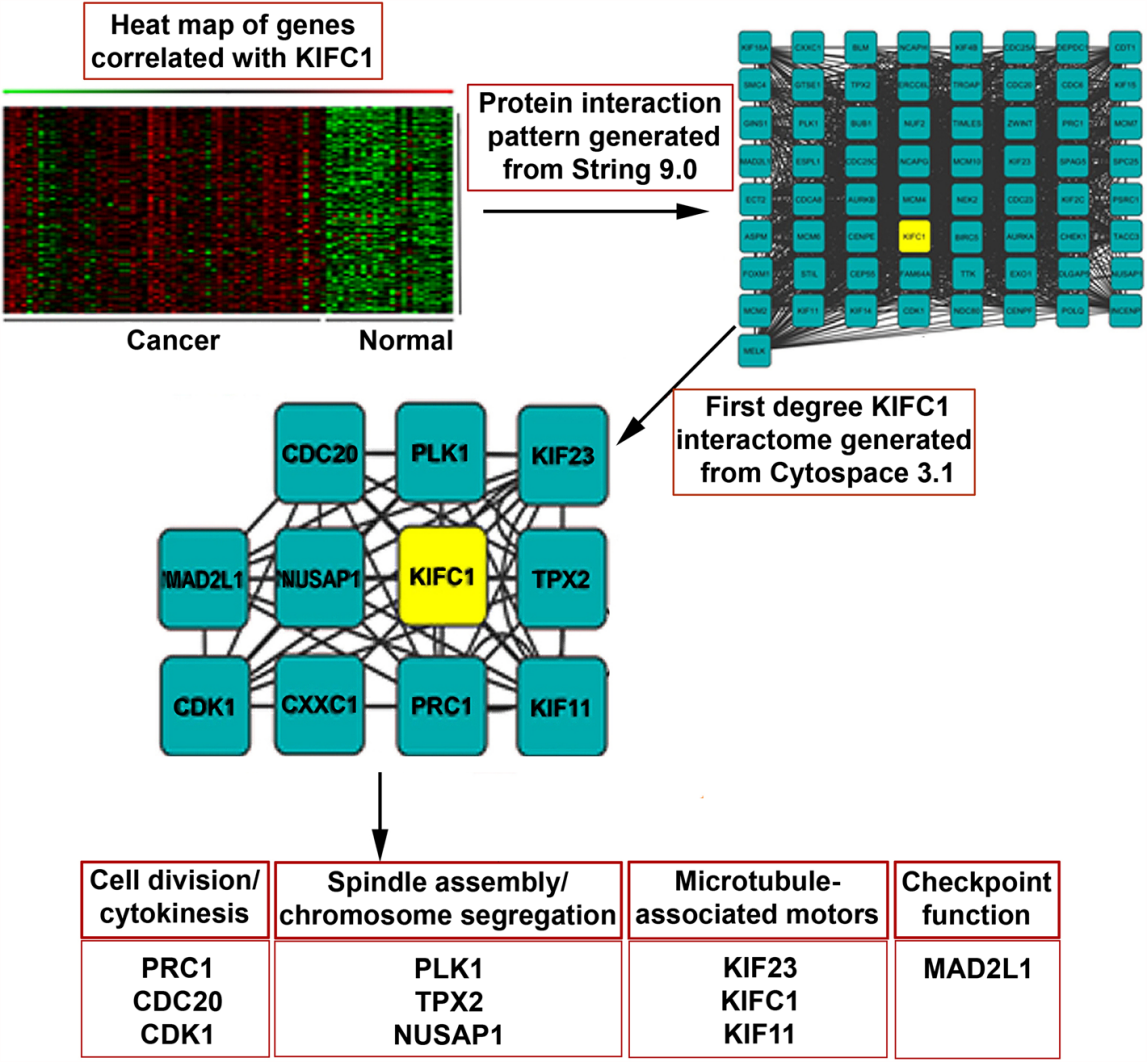
**Pathway analysis for KIFC1.** Interactome of high and low correlating genes and their interactions with KIFC1. Pathways associated with first degree neighbors of KIFC1 protein.

We next sought correlations between 22276 Affymetrix genes and KICF1 in cancer and normal ovarian samples using Pearson’s correlation coefficient. Genes with a correlation value of >0.5 and between -0.1 and -0.5 were fed in the Ingenuity pathway analysis (IPA) tool to identify cell cycle and related pathways (Table 2). A detailed list of all associated pathways is provided in Additional file 1: Table S1. Interactions with KIFC1 protein were explored with STRING 9.1. Proteins for genes which had highest and lowest correlations with KIFC1 were fed into STRING 9.1 [22] to obtain confidence values for their interactions, which were then fed into Cytoscape 3.0 [23] for development of an interactome (Figure 4). We found Mitotic arrest deficient-like 1 (MAD2L1), Polo-like kinase 1 (PLK1), Cell division cycle 20 (CDC20), cyclin-dependent kinases (CDK1), Nucleolar and spindle associated protein 1 (NUSAP1), Protein regulator of cytokinesis 1 (PRC1), Kinesin family member 11 (KIF11), Targeting Protein For Xklp2 (TPX2), and Kinesin family member 23 (KIF23) as first degree neighbors with KIFC1 (Figure 4). The confidence value for each of these interactions is given in Table 3.

**Table 2:** Pathways and genes involved with KIFC1 and its correlated genes

**Pathways associated**	**No of genes**	**% of genes involved**
Cell cycle	54	73
Cell cycle checkpoint	13	17.6
Cell division	35	47.3
Cell proliferation	15	20.3
Chromosome condensation	6	8.1
Chromosome segregation	16	21.6
M phase	45	60.8
Microtubule cytoskeleton	36	48.6
Mitotic cell cycle	45	60.8

**Table 3:** Confidence values for KIFC1 and respective interactions

**Interaction(s)**	**Confidence value**
KIF23	0.622
PRC1	0.626
MAD2L1	0.638
PLK1	0.646
CDK1	0.671
TPX2	0.677
CDC20	0.717
NUSAP1	0.734
KIF11	0.806

## Discussion

Over the recent years, the role of KIFC1 in centrosome clustering in cancer cells with supernumerary centrosomes has been well recognized. Our present study shows that both low- and high- grade ovarian tumors display CA. This finding is consistent with a previous report showing presence of CA in ovarian tumors in a stage-dependent manner [13]. Interestingly, we found that while low-grade tumors display a higher proportion of mitotic cells displaying multipolar mitosis compared to high-grade ones, centrosome clustering was almost an exclusive feature of high-grade tumors compared to low-grade ones. As part of the tumor evolution agenda, we rationalize that when tumors are low-grade, multipolar mitosis reflects their proclivity to undergo aberrant mitoses and subsequent cell divisions. Perhaps multipolar mitosis enables them to maintain low-grade aneuploidy which fosters tumor growth and progression. On the other hand, maintaining enhanced centrosomal clustering as in the case of high-grade tumors may serve cancer cells by helping them attain a more aggressive phenotype [2]. We believe that centrosome clustering confers cancer cells with cytoskeletal advantages that may enhance cell polarization, Golgi-dependent vesicular trafficking, stromal invasion, and other aspects of metastatic progression [2].

Since high-grade ovarian cancers display a high degree of centrosomal amplification coupled with a configuration that keeps them in a “bundled” or clustered state, it is reasonable to expect that they overexpress proteins that will aid in centrosome clustering. To this end, we examined the expression of KIFC1, a known centrosome clustering molecule, and evaluated its prognostic power in ovarian cancer. We found KIFC1 levels to be significantly higher in ovarian cancer compared to normal ovarian epithelia. It is noteworthy that increasing KIFC1 levels were associated with increasing grades. This is especially important from a clinical viewpoint as the likely course of disease could be predicted accurately by measuring KIFC1 expression in different grades. Even though KIFC1 levels in both primary and metastatic tissue were significantly higher compared to normal tissue, KIFC1 expression between matched primary and metastatic ovarian carcinoma were not different. We speculate that the primary tumor expressed KIFC1 to a high enough level to cause epithelial to mesenchymal transition (EMT) that marks the beginning of the metastatic journey. Thus, it is likely that KIFC1 may serve to be a marker for metastatic onset. The overall survival was also lower in patients with high KIFC1 expression, implying its value as a prognostic biomarker in ovarian cancer. We further delineated the proteins that might be interacting with KIFC1 gene and found that many of these potentially interacting proteins were cell-cycle related genes. KIFC1 is a kinesin involved in various cellular processes such as mitotic spindle assembly [24], centrosome clustering [25,26], and vesicle transport [27] in cancer cells. Thus, its interaction with an array of cell cycle-specific proteins is ostensible. Nevertheless, these data implicate the role of KIFC1 in the regulation of cell-cycle and interaction with these proteins. It is likely that KIFC1 has clustering-independent role in cancer cells which require further investigation.

## Conclusion

Taken together, we demonstrate that ovarian cancers display amplified centrosomes, a feature of aggressive tumors. To cope up with the abnormal centrosomal load and at the same time circumvent mitotic catastrophe, ovarian cancer cells upregulate genes like KIFC1 that are known to induce centrosome clustering, a “tactic” that tumor cells have evolved to execute mitosis in a pseudo-bipolar state. Our data compellingly underscores that KIFC1 can be a prognostic biomarker in ovarian cancers. Our interactome data have discovered some “purely” novel potential binding partners based upon pathway connectivity, which merit further screening and investigation to shed more light into the possibly clustering independent roles of KIFC1.

## Material and methods

### A) In silico analysis of KIFC1 gene expression

#### ***A.I. Data collection***

One channel micro array data were collected from Gene Expression Omnibus (GEO) database and Cancer Genome Atlas (TCGA). List of gene identities (ID’s) is given in Table 4.

**Table 4:** List of Gene ID

**Normal samples GEO Series ID**	**Cancer samples GEO Series ID**
GSE14407, GSE18520 (N = 38)	GSE20565, GSE14764, GSE12418, GSE41498, GSE9890, GSE9891 (N = 494)
	https://tcga-data.nci.nih.gov/datareports/aliquotIdBreakdownReport.htm (N=595)

The gene expression data for the fourteen matched sets of primary and metastatic (omental) samples were procured from a recently published paper from John McDonald’s group [20].

#### ***A.II. Data pre-processing***

One channel micro array data was Mas5.0 normalized [28], and was further taken for processing.

#### ***A.III. Identification of KIFC1 gene expression***

Logarithm to the base 2 transformed KIFC1 expression levels of ovarian cancer patients were extracted from the TCGA and GEO patients and compared to their normal pairs. Identification of KIFC1 expression levels for 14 primary and secondary ovarian cancer samples was done following AI, AII, and AIII protocols.

#### ***A.IV. KIFC1 gene expression and associated clinical outcome information***

Associated grade, stage and clinical information for 468 patients from GEO database were plotted for KIFC1 gene expression values.

### B) Interactions of KIFC1 protein

KIFC1 gene was correlated with 22277 affymetrix probe id’s, genes with a correlation value of >0.5 and between -0.1 and -0.5 were fed in the IPA tool to identify pathways associated. Proteins and their interactions amongst them with confidence values were extracted from STRING 9.1, and inputted in Cytoscape 3.0 for building interactomes.

### C) tissue specimens

In this study, a total of 14 human serous ovarian cancer specimens and their normal adjacent were procured from Northside Hospital (Atlanta, GA). There were 7 each of low- and high- grade cancer tissues.

### D) Immunofluoresence

Slides were first deparaffinized by baking in oven at 60°C for 2 h followed by 3 xylene baths. Rehydration was then performed in a series of ethanol baths (100%, 90%, 75% and 50%). Antigen retrieval was achieved by citrate buffer (pH 6.0) in a pressure-cooker (15 psi) for 3 min. Primary antibodies (1:2000 dilution) were incubated with the slides for 45 min at 37°C. The cells were washed 10× with PBS at room temperature before incubating at 37°C with a 1:2000 dilution of conjugated secondary antibodies. Cells were washed 5× with PBS and then mounted with Prolong-Gold antifade reagent that contained DAPI (Invitrogen).

### E) Statistical analysis

Statistical analysis was performed using Student’s t-test and the criteria for statistical significance was p < 0.05.

## Competing Interests

The authors declare that they have no competing interests.

## Authors’ contributions

SP and SD carried out data analysis and assisted in the writing of the manuscript; VP carried out the immunofluoresence staining; PR, NB and AO assisted in data analysis; RO carried out the statistical analysis; GC and RA designed the study and assisted in the writing of the manuscript. All authors read and approved the final manuscript.

## Supplementary Material

Additional file 1 List of Pathways Associated with KIFC1. Click here for file
